# Spectral sensitivity and color discrimination of *Euxesta eluta* and *Chaetopsis massyla* (Diptera: Ulidiidae)

**DOI:** 10.1371/journal.pone.0346423

**Published:** 2026-04-03

**Authors:** Sandra A. Allan

**Affiliations:** United State Department of Agriculture, Agricultural Research Service, Center for Medical, Agricultural and Veterinary Entomology, Gainesville, Florida, United States of America; National Institute of Agricultural Research - INRA, MOROCCO

## Abstract

Cornsilk flies, *Euxesta eluta* and *Chaetopsis massyla* (Diptera: Ulidiidae), are serious economic pests affecting sweet corn production in Florida. As a basis for development of enhanced trapping strategies, the sensory and behavioral basis for response to color was examined. Using electroretinograms, spectral sensitivity curves for both species revealed broad curves with a peak in the UV (350 nm) and the green region (500–550 nm) of the spectrum. Curves for males and females of each species were relatively similar in shape. Using pigment templates, the measured curve for *E. eluta* was matched by pigment combinations with maximum sensitivity at 350, 430, 500 and 560 nm in a ratio of 25:21:25:29. Similarly, the curve for *C. massyla* was best matched by pigments with maximum sensitivity at 350, 430, 500 and 560 nm in a 28:18:27:27 ratio. Laboratory behavioral assays were conducted to evaluate if attraction occurred in response to the color or the brightness of a target. Attraction responses were evaluated in paired tests to blue, green and yellow cards paired with gray cards of matching brightness. Despite relatively similar visual pigments, the two species differed greatly in their behavioral attraction. *Euxesta eluta* was only attracted to yellow cards at low or high brightness levels, to mid-level bright blue cards and not attracted to green cards at any brightness level. In contrast, *C. massyla* was highly attracted to yellow and green cards compared to gray cards of the same brightness but avoided blue cards. These differences in response to color and brightness are important for interpretation of surveillance results as well as development visual traps targeted for these species.

## Introduction

Cornsilk flies (Diptera: Ulidiidae) (Acalyptratae, Tephritoidea) are major pests affecting sweet corn production in warm temperate and tropical areas in North, Central and South America [1–3]. The preference of these flies to oviposit on cornsilk and the subsequent larval development within the corn ear is responsible for significant crop loss [4]. In Florida, where sweet corn production has been valued at nearly $150M [5], damage can exceed >20% of ears in a field, potentially resulting in rejection at packing houses [4,6]. Most of the damage caused in Florida is associated with *Euxesta eluta* Loew, *Chaetopsis massyla* (Walker) and *Euxesta stigmatias* Loew [3].

Host plant attributes such as odor and vision are presumably involved in the location of potential host plants by cornsilk flies [7,8]. Evaluations of potential volatile attractants for location of host plants from both laboratory [7] and field studies [1,6,9] revealed attraction to protein-based foods. Odor-based yellow McPhail traps used in a survey in Brazil [1] provided control of two *Euxesta* species in an experimental 0.8 ha plot [9]. Surveillance, however, in commercial scale production currently relies on field observations as surveillance traps have yet to be optimized. Additional insights into the role of visual cues can provide an important component towards development of surveillance strategies for these flies.

The role of color in insect vision involves perception of photopigments and behavioral response to color stimuli as well as neuronal processing. Color vision requires the demonstration of at least two different spectral receptor types, discrimination of different colors independent of the intensity or brightness of the stimuli as well as entailing color opponency or comparison from at least two or more spectral types of photoreceptors to differentiate wavelengths [10–12]. The basis of any color choice behavior is the spectral type of photoreceptor present [13,14]. Most insects have photoreceptors with maximum sensitivity in the ultraviolet (350 nm), blue (440 nm) and green (530 nm) portion of the spectrum [12,13,15]. Species, however, may differ in the maximum sensitivity of individual photopigments and numbers and proportions of pigments present [12,13]. Examination of the electrophysiological responses to different wavelengths through electroretinograms can provide insight into the photopigments that may provide the basis for behavioral responses. While greater sensitivity in a particular region of the spectrum may not necessarily convey behavioral attraction to that color, it may serve other purposes such as providing contrast to enhance attributes such as color or movement detection independent of brightness [16–18]. Response to visual stimuli can be a function of both and/or the dominant wavelength (hue) and the brightness (intensity) of the stimuli or both. For instance, a behavior such as attraction can be elicited by a specific wavelength (hue) but be dependent on the brightness of the stimuli and this constitutes wavelength-specific behavior [17]. In contrast, attraction occurring to a wavelength (hue) independent of stimuli brightness is considered brightness-independent color discrimination [13,17].

Attraction to visual attributes such as hue, shape, pattern, and background contrast have been reported for both *E. eluta* and *C. massyla* [8,19]. While *E. eluta* showed broad attraction to yellow orange, and yellow green, both studies also reported attraction to blue traps [8,19]. In contrast, *C. massyla* reported to be strongly attracted to both yellow and yellow green traps with lower attraction to orange or blue traps [8]. In these studies, brightness between colors was not excluded as a factor for attraction and further studies are needed to clarify the role of hue in attraction.

As a basis for understanding color behavioral choice in these insects, the physiological basis for color detection in *E. eluta* and *C. massyla* was examined. Behavioral assays further examined the potential for color discrimination independent of brightness.

## Materials and methods

### Insect rearing

Two cornsilk fly species, *E. eluta* and *C. massyla*, were maintained in colony on a corn kernel/agar diet along with fresh green pepper slices [7,20]. Adults were maintained in screen cages in environmental chambers at 27^o^C, 60–80% relative humidity and under a 16:8 L:D photoperiod. Flies were provided with 10% sugar water *ad lib*. Adult flies used for electrophysiology were 10–14 days old.

### Electrophysiological techniques

Electroretinograms (ERG) to examine the spectral sensitivity of the compound eyes were produced by the flash method [21]. Individual flies were gently aspirated, wings held with forceps and then wings and legs were gently pressed into softened sticky wax (Cenco, Central Scientific Company, Chicago, IL). Melted wax was used to position the head with care not to coat the eyes or spiracles. Electrolytically-sharpened tungsten wires (0.127 mm, A-M Systems, Carlsburg, WA) were used as electrodes. The indifferent electrode was placed in the thorax and the recording electrode inserted in a central position of the left eye where a light beam was projected. The light was delivered through a UV-VIS quartz optic fiber (6.3 mm diam, Dolan-Jenner Industries, Boxborough, MA) and positioned with a micromanipulator 1 cm from the eye surface. All recordings were conducted in a grounded Faraday cage covered with blackout cloth in a dark room (27 – 28^o^C, 60–72% relative humidity).

Light from a halogen lamp (EXR 300W/82V, OSRAM) was transmitted through a narrow bandpass interference filter (10 nm) (Edmund Industrial Optics, Barrington, NJ) to produce the monochromatic light stimulus. A series of fifteen different filters were used and represented wavelengths between 340 and 700 nm. Neutral density filters were used as needed to adjust light flux as needed to produce quantal stimulation at each wavelength. Neutral density filters consisted of a circular variable neutral density filter (Edmond Optics, Barrington, NJ) (0.04–4 OD) placed in a 360^o^ wheel mount with stops at every 10^o^, as well as additional neutral density filters ranging from 0.5–4.0 OD (5 cm x 5 cm, Wratten gelatin filters; Eastman Kodak Company, Rochester, NY). A 200 ms pulse of light controlled through a manual shutter provided light simulation with at least one-minute intervals of darkness between light flashes.

An amplifier (10x) associated with the electrode (EAG COMBI 10X, Syntech, Hilversum, The Netherlands) in conjunction with an additional amplifier (10x) (Syntech Autospike IDAC) provided a total of 100x magnification. The signal was captured using Syntech EAG software (ver. 2.4 Syntech, Kirchzarten, Germany) on a computer. Calibration of the optical system for all combinations of interference and neutral density filters was conducted using a concave grating spectroradiometer (Black Comet CXR-SR, StellarNet Inc, Tampa, FL) and quantal flux calculated.

Intensity-response curves of the amplitude of the positive on-response of the ERG (mV) at different light intensities over a range of wavelengths (380, 430, 470, 520, 580 nm) were obtained. Response voltage plotted versus log of light intensity (uW/cm^2^/s) (V-*log*I) produced intensity-response curves with a log-linear function and minimum criterion response obtainable across the range of wavelengths determined. Ten individuals of each sex and species were evaluated.

For each preparation, a preliminary stimulus at 550 nm was provided and if it resulted in a typical ERG response, *i.e.,* a standard negative waveform, then the fly was dark-adapted for 1 hr to allow photopigments to regenerate and maximize sensitivity. At 550 nm eyes were exposed to very low light intensity and intensity increased until a response of 1 mV (half the criterion response) was obtained. This intensity differed between individual flies and the response to the selected intensity at 550 nm was noted at the beginning, middle and end of a recording session to determine if the preparation had degraded (in which case, the fly was discarded). Measurements of the voltage response at each wavelength were initiated with low light intensities and increased in intensity until the criterion response was reached. Each fly was tested at all 15 selected wavelengths (filters) (lowest to highest nm), three readings taken that were averaged.

### Spectral sensitivity

The spectral sensitivity curves for the eyes were calculated as the reciprocal of the quantum flux required to elicit the criterion response for each of the 15 wavelengths tested. The number of photons that produced the criterion response at each wavelength was determined from a linear regression of neutral density against the number of photons x10^12^ with the inverse of the number of photons providing the spectral sensitivity values. The sensitivity of the curve was denoted by the equation *S*(*λ*) = Σ *α*_*i*_ (*λ*), with S = spectral sensitivity and *α*_*i*_ (*λ*) = the sensitivity of the alpha band at each wavelength [22].

Spectral sensitivity curves were calculated from the mean of ten flies of each species and sex. Differences in absolute sensitivity between individual insects were removed by normalizing individual curves then averaging [23]. Curves were calculated first as means for each sex for each species for comparison between sexes, then data for each species combined for comparison between species. These curves represented the total response from the portion of the eye where it was recorded and represented contributions from numerous individual photopigments.

The spectral sensitivity of an individual photopigment is characterized by its peak of sensitivity or λ_max_ [24] as a function of the absorption spectrum of the particular individual visual pigment [12]. The measured spectral sensitivity curves from the eyes of the flies were compared to theoretical visual photopigment absorption curves for individual photopigments with different α-peak maxima (λ_max_). The theoretical curves were derived using rhodopsin absorption templates based on Stavenga [25] to provide curves below 350 nm and modified to fit Govardovskii et al. [24] pigment templates for longer wavelengths [26]. Fly spectral sensitivity curves were initially compared to single visual pigment templates. Subsequently iterative mixtures of pigment templates with different λ_max_ absorption curves were combined in different ratios to obtain an optimal fit to the measured spectral sensitivity.

### Behavioral assays

Both the color hue and brightness of a stimulus can affect attraction of an insect [12]. Assays comparing innate attraction to colored cards paired with gray cards of equal brightness were conducted to examine brightness-independent color discrimination in cornsilk flies. Colors selected for comparisons included blue, green and yellow pigments with maximum reflectance at 430, 515, and 570 nm, respectively, that were relatively close to the observed pigment maxima from the electroretinogram study. Assays were conducted in white screened cages (60 x 60 x 180 cm) (Megaview Science, Taichung, Taiwan) containing two cards, one colored and one gray but of the same brightness of reflected light. Landing responses of flies based on numbers attached to the sticky surface were compared for each card. Visual distraction was reduced with a white cardboard structure and fabric surrounding the cages. Lighting was provided by 250-watt metal halide lamps lit at least 20 min before tests started and provided 5767 lux at the mid-height of the cards. For each test 35–40 flies (10–14 days old) were collected by aspiration and released in the center floor of each cage. The sex ratio of these collections was roughly 50:50 male:female. Assays were initiated at 7–8 am, conducted for 4 hr then cards collected, remaining flies collected, and all flies sexed and counted. Preliminary assays with hourly collections of flies indicated no significant increase in collections after 4 hr. Assays were conducted at 27 ± 1^o^ C. Within assay cages, two cards (one colored and one corresponding gray) were placed equidistantly along the back wall with the sticky side of each card facing into the cage. Preliminary tests indicated no positional bias between cages and trap positions. However, the position of each card, left or right, was still noted for each replicate and placement switched in subsequent assays. Assays were repeated in four cages on four separate days.

Cards (10 x 10 cm) were prepared from acetate sheets initially painted with a base coat of white titanium dioxide acrylic primer on one side. Acrylic paint mixtures were formulated with titanium white, mars black, cadmium yellow medium hue, permanent green light, and cobalt blue hue (Galeria Acrylic, Winsor & Newton, London, UK). For each paint hue, cards were painted with 100% colored paint and four paint dilutions with white paint that ranged from 10–80% colored paint. Because pigment concentration varied between different colored formulations of paint, dilutions with white paint differed between colors but selected to provide an even range of brightness for each color. Corresponding gray mixtures were then formulated to produce a gray paint similar in brightness to each color mixture. Matching mixtures were determined by using reflectance values (uwatts/cm^2^/sec) from radiometer readings between 300 and 700 nm under a standard tungsten light. The painted side of each card was covered with clear double-sided sticky sheet to collect flies (Alpha Scents, West Linn, OR) and a wire attached to hang cards in the cages.

Data for two choice assays were calculated as the % of flies collected on cards and were analyzed for normality by the Shapiro-Wilk test and for homogeneity of variance by Levene’s test (α = 0.05) for homogeneity of variance and Shapiro-Wilk test (SigmaStat, Systat, San Jose, CA, USA). For normal data, parametric tests, ANOVA, t-test) were used and for non-normal data, non-parametric (Kruskal-Wallis) tests were used. Assay data were collected over four dates and within each color/gray comparison, statistical comparisons within each color/gray comparison were made between dates (*P* = 0.05) which revealed no significant differences among dates (*P* > 0.05), so data from all four assay dates were pooled. Similarly, comparisons made of responses of each sex were similar and data were combined for analysis and presentation.

### Light and reflectance measurements

A concave grating spectrometer (UV-VIS Black Comet, StellarNet Inc., Tampa, FL, USA) with quartz light guides was used for light and reflectance measurements. Cards were illuminated with a deuterium/tungsten/halogen light source (StellarNet, Inc. Tampa, FL, USA) and reflected light measured. Measurements were standardized with a white halon standard and a dark (no light) standard.

## Results

**Fig 1:**
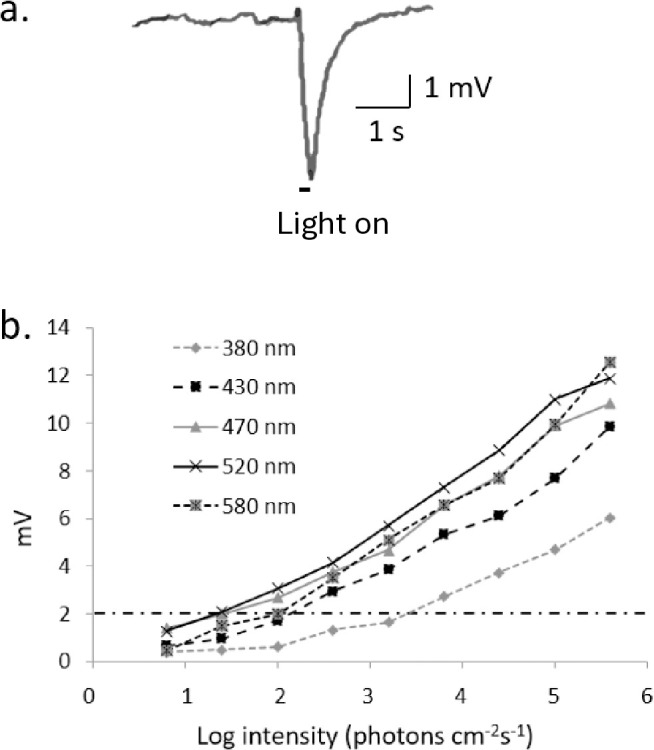
Electroretinogram responses of *Euxesta eluta.* **A. Typical waveform, B. Intensity-response function (V/log I) at selected waveforms for a representative male *E. eluta*.** The selected criterion response (2 mV) is designated by the dotted horizontal line.

A typical dipteran waveform observed in cornsilk flies was biphasic (Fig 1A) and consisted of a positive on-response with onset of light and a negative deflection lasting the duration of illumination. For all wavelengths examined, standard log linear intensity-response curves were generated, and representative responses are presented in Fig 1B. These curves are broadly parallel over a range of wavelengths and a criterion response of 2 mV, which was obtained across the range of wavelengths, was selected for the spectral sensitivity studies.

**Fig 2:**
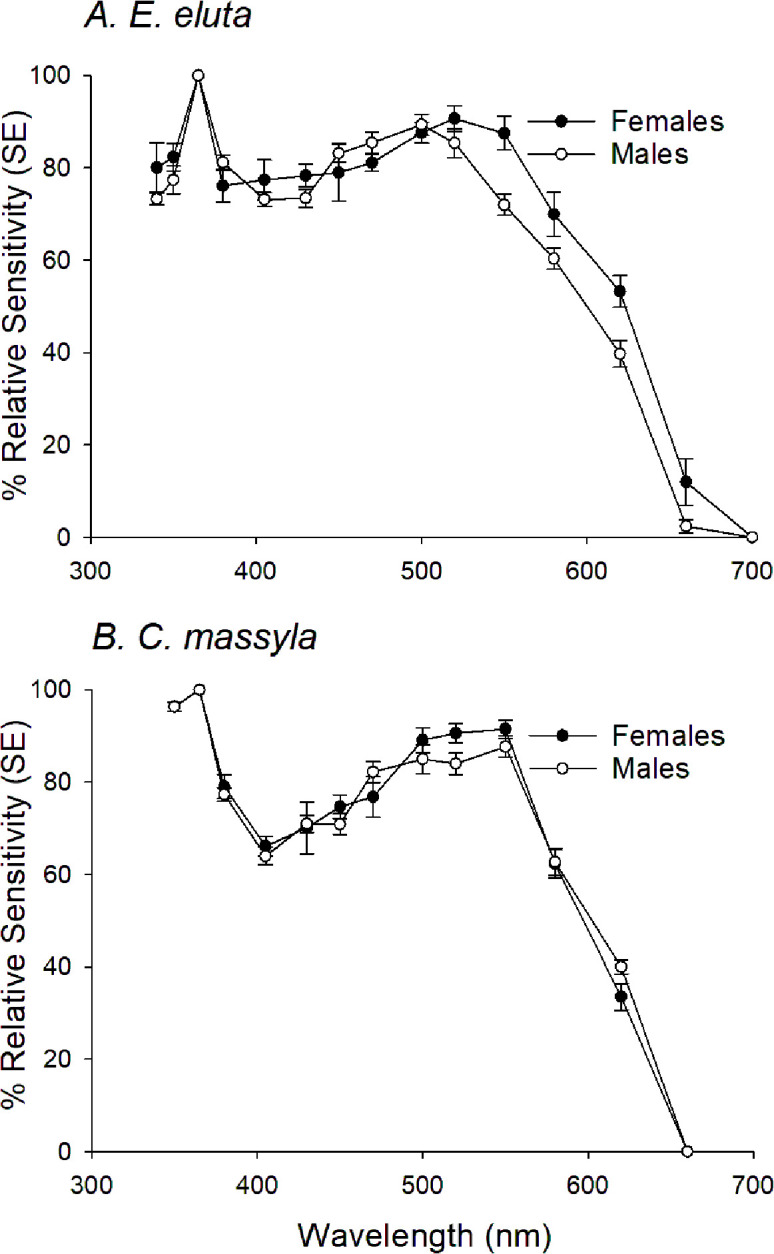
Spectral sensitivity curves for female and male: A. Euxesta eluta, and B. Chaetopsis massyla.

**Fig 3:**
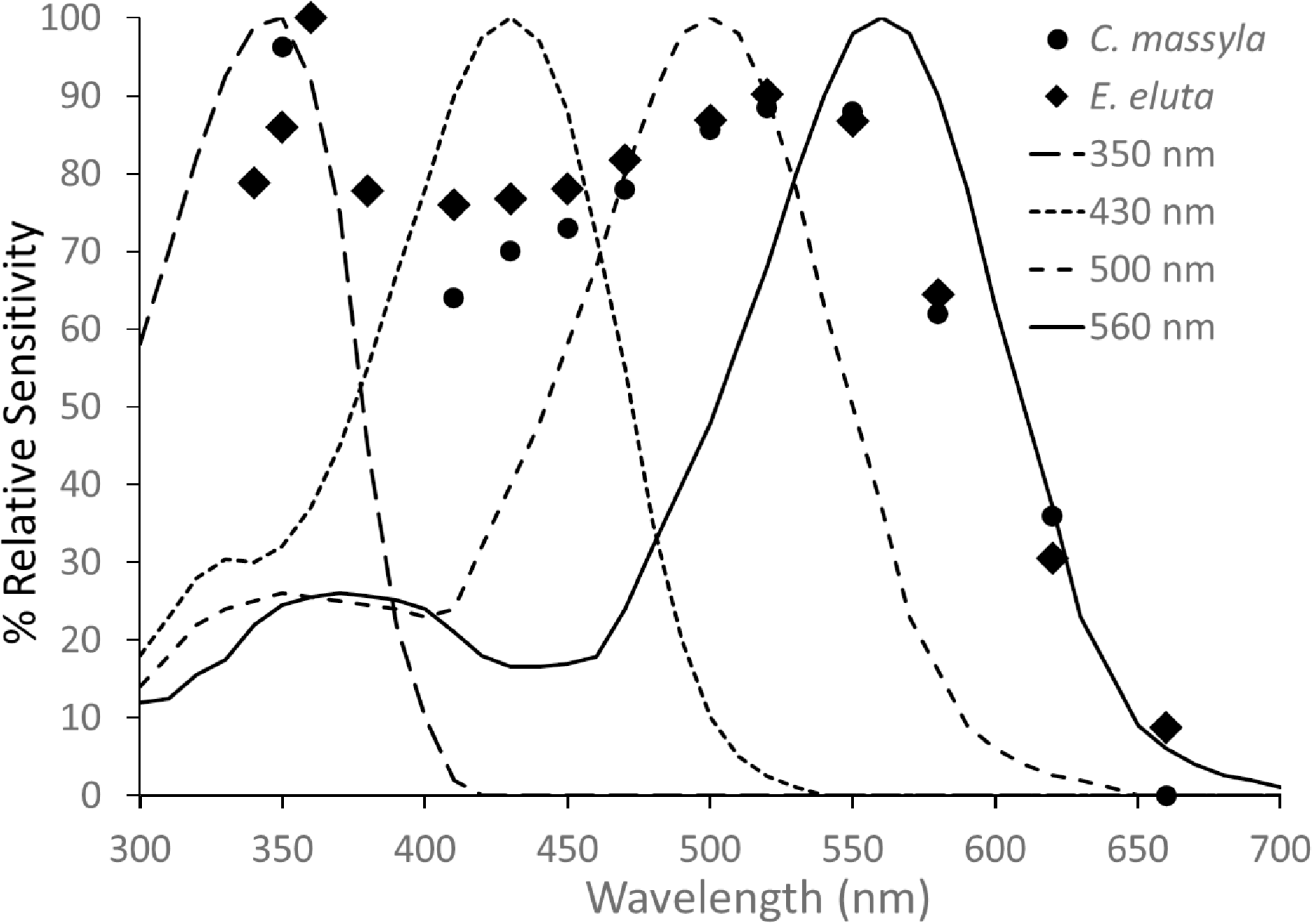
Absorbance curves of individual pigments modelled using visual pigment templates and averaged spectral sensitivity values for *E. eluta.*

**Fig 4:**
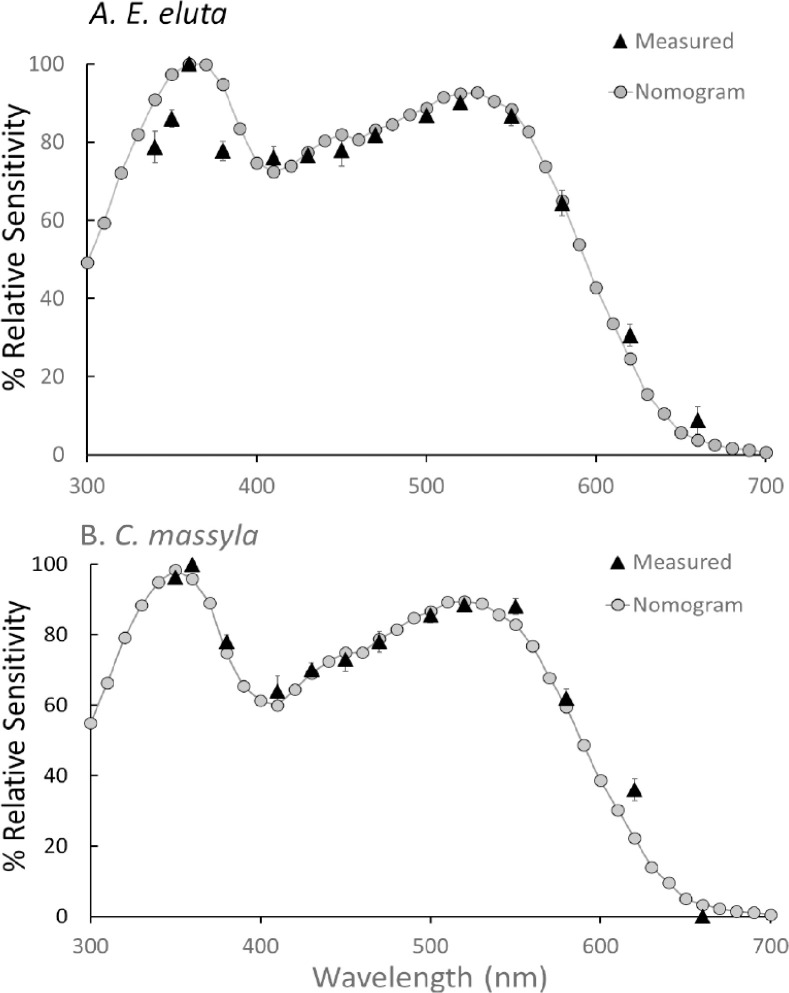
Matching of the average spectral sensitivity curves for *Euxesta eluta* (A) and *Chaetopsis massyla* to mixtures of photopigments (nomograms). The spectral sensitivity curve for *E. eluta* was fit with a mixture consisting of 355:430:500:560 nm in a ratio of 25:21:25:29. The curve for *C. massyla* was fit to a mixture consisting of 350:430:500:560 nm in a ratio of 28:18:27:27.

The spectral sensitivity curves for *E. eluta* and *C. massyla* were similar in shape with a peak in the UV (about 350 nm) followed by a decrease in sensitivity in high UV wavelength and blue regions and then a broad peak with maxima about at 520–550 nm (Fig 2A, B). Curves for males and females of *E. eluta* were very similar except a slight decrease in sensitivity of male *E. eluta* at higher wavelengths (Fig 2A), while those for male and female *C. massyla* were very similar (Fig 2B). Sensitivity curves for each species were averaged then used for subsequent pigment matching. Curves for individual theoretical pigments with λ_max_ of 350, 430, 500, and 560 nm failed to account for the broad sensitivity curve measured for both species (Fig 3). From iterative combinations, curves comprised of several pigments were found to best fit by eye to the measured spectral sensitivity curves (Fig 4). The measured curve for *E. eluta* was matched by a combination of 350, 430, 500 and 560 nm in a ratio of 25:21:25:29 (Fig 4A). Similarly, the curve for *C. massyla* was best matched by a combination of 350, 430, 500 and 560 nm in a 28:18:27:27 ratio (Fig 4B).

**Fig 5:**
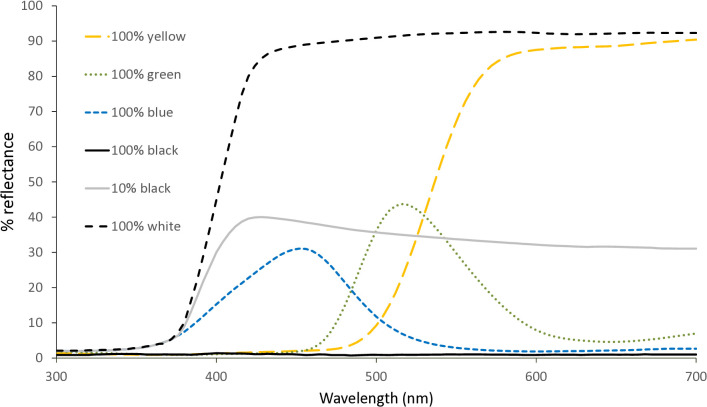
Reflectance from cards painted with 100% blue, green and yellow paints. For comparison, reflectance from 100% black, 100% white and 10% gray are also presented.

Paints used represented the broad classes of colors considered for attraction and included blue (max 430 nm), green (max 515 nm) and yellow (max 570 nm). Reflectance curves of colored cards are presented in Fig 5**.** Assays were conducted with both male and female flies and as there was no difference in response between the sexes, responses were combined (two tailed two sample t-test, *P* > 0.05). The overall proportions of released flies collected in total (colored card and gray card) differed between species (S1Table) with lower percentages of released *E. eluta* collected (34.11 ± 1.85) compared to *C. massyla* (70.70 ± 2.32) (*t* = 1.70, df = 27, *P* < 0.001). Percen*t*age collections of *E. eluta* did not differ between colors tested (*F* = 1.55, df = 2,14, *P* = 0.25), however significantly more *C. massyla* were collected in assays with yellow (78.36 ± 3.85) compared to assays with blue (64.72 ± 3.25) and both were similar to assays with green (69.01 ± 2.67) (F = 4.47 ± df = 2, 14, P = 0.035).

**Fig 6:**
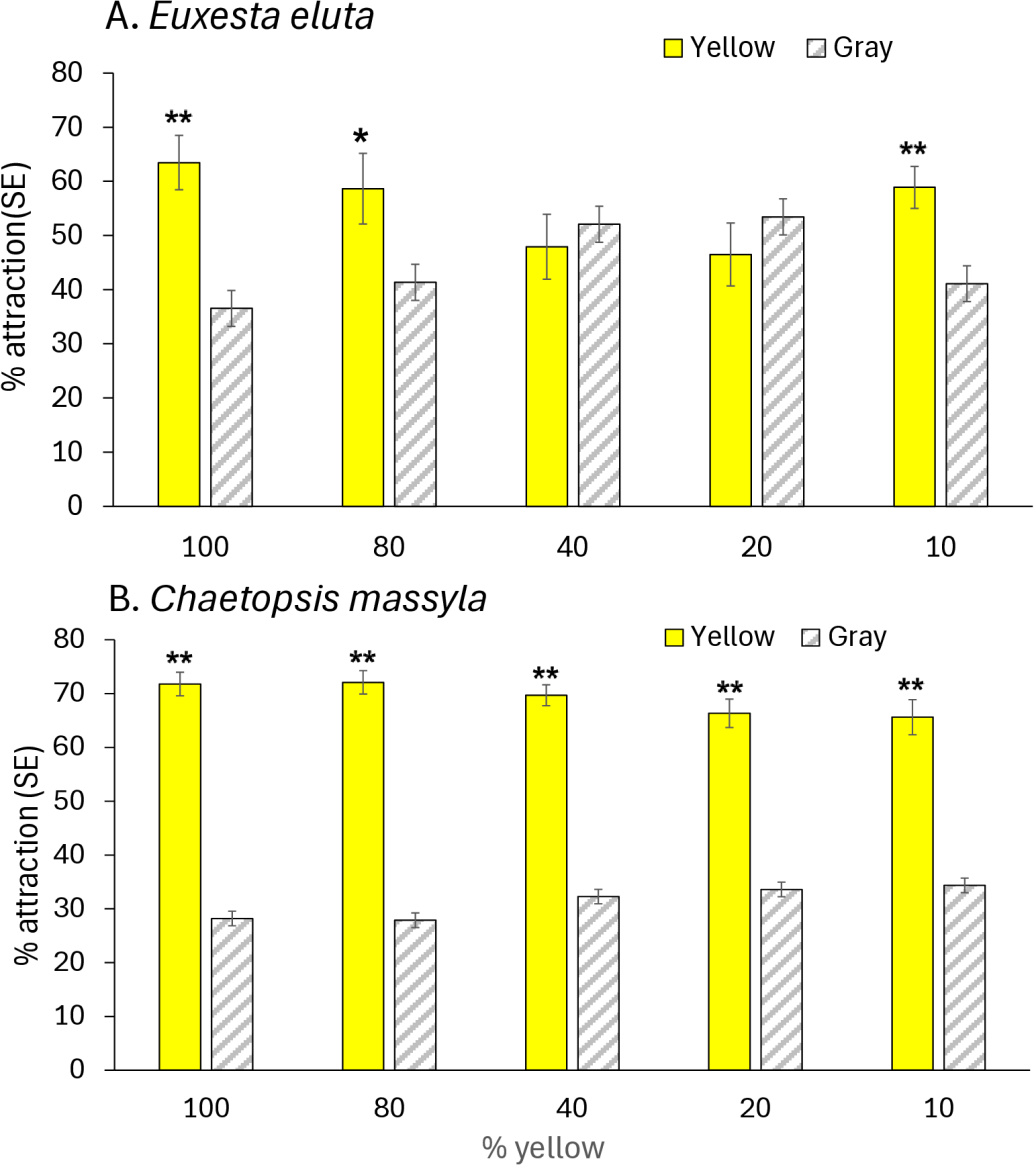
Attraction of *Euxesta eluta* (A) and *Chaetopsis massyla* (B) in paired comparisons with a yellow card and a gray card matching in brightness. The different levels of brightness of yellow are represented by a range of yellow pigment (10-100%) combined with white pigment. Gray cards produced by combination of black and white pigment matched each yellow mixture in brightness. Asterisks (*) above paired columns denote statistically significant differences at *P* < 0.05, double asterisks (**) denote significant differences at *P* < 0.001 and no asterisks represent no significant differences. N = 16.

**Fig 7:**
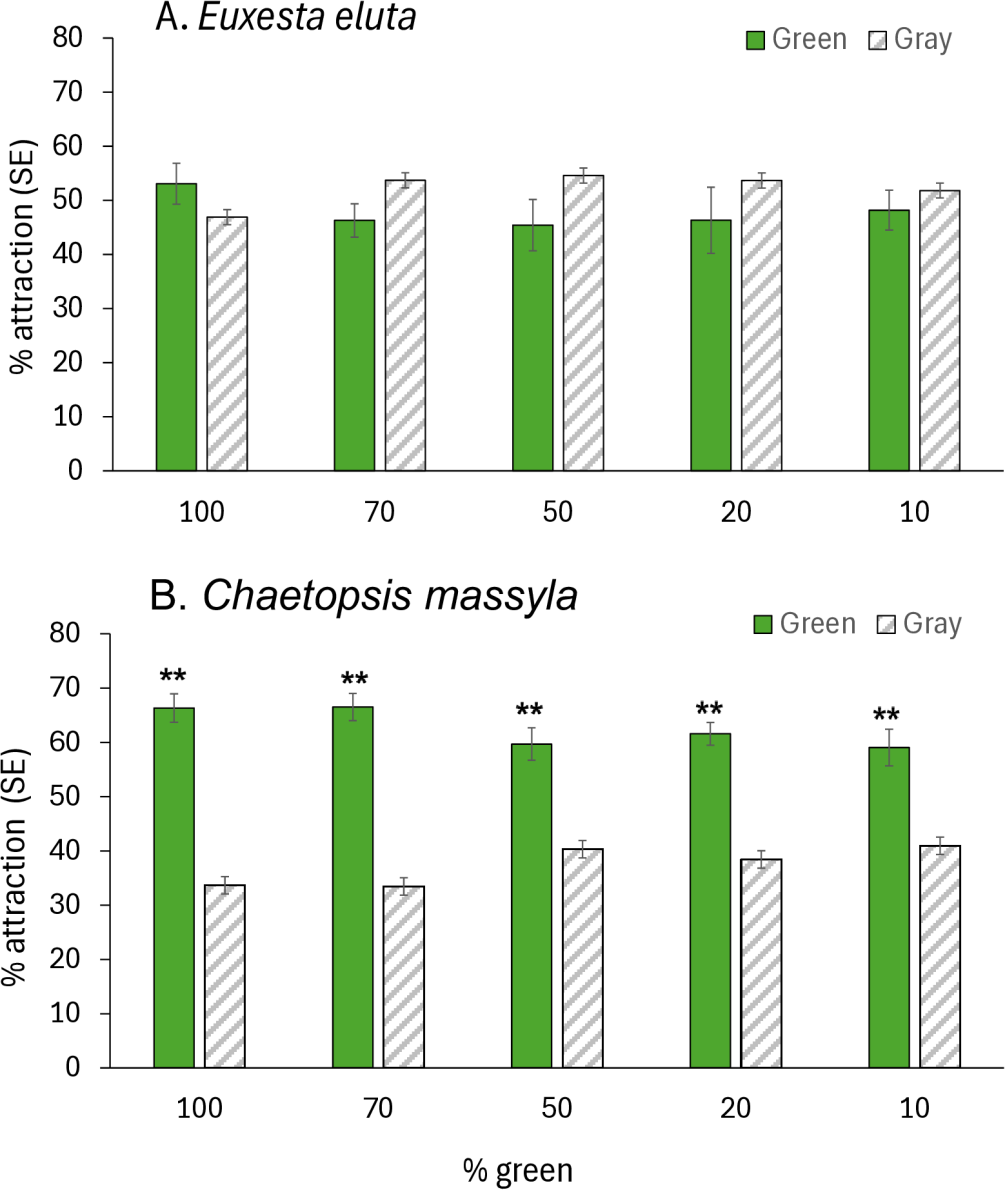
Attraction of *Euxesta eluta* (A) and *Chaetopsis massyla* (B) in paired comparisons with a green card and a gray card matching in brightness. The different levels of brightness of green are represented by a range of green pigment (10-100%) combined with white pigment. Gray cards produced by combination of black and white pigment matched each green mixture in brightness. Asterisks (*) above paired columns denote statistically significant differences at *P* < 0.05, double asterisks (**) denote significant differences at *P* < 0.001 and no asterisks represent no significant differences. N = 16.

**Fig 8:**
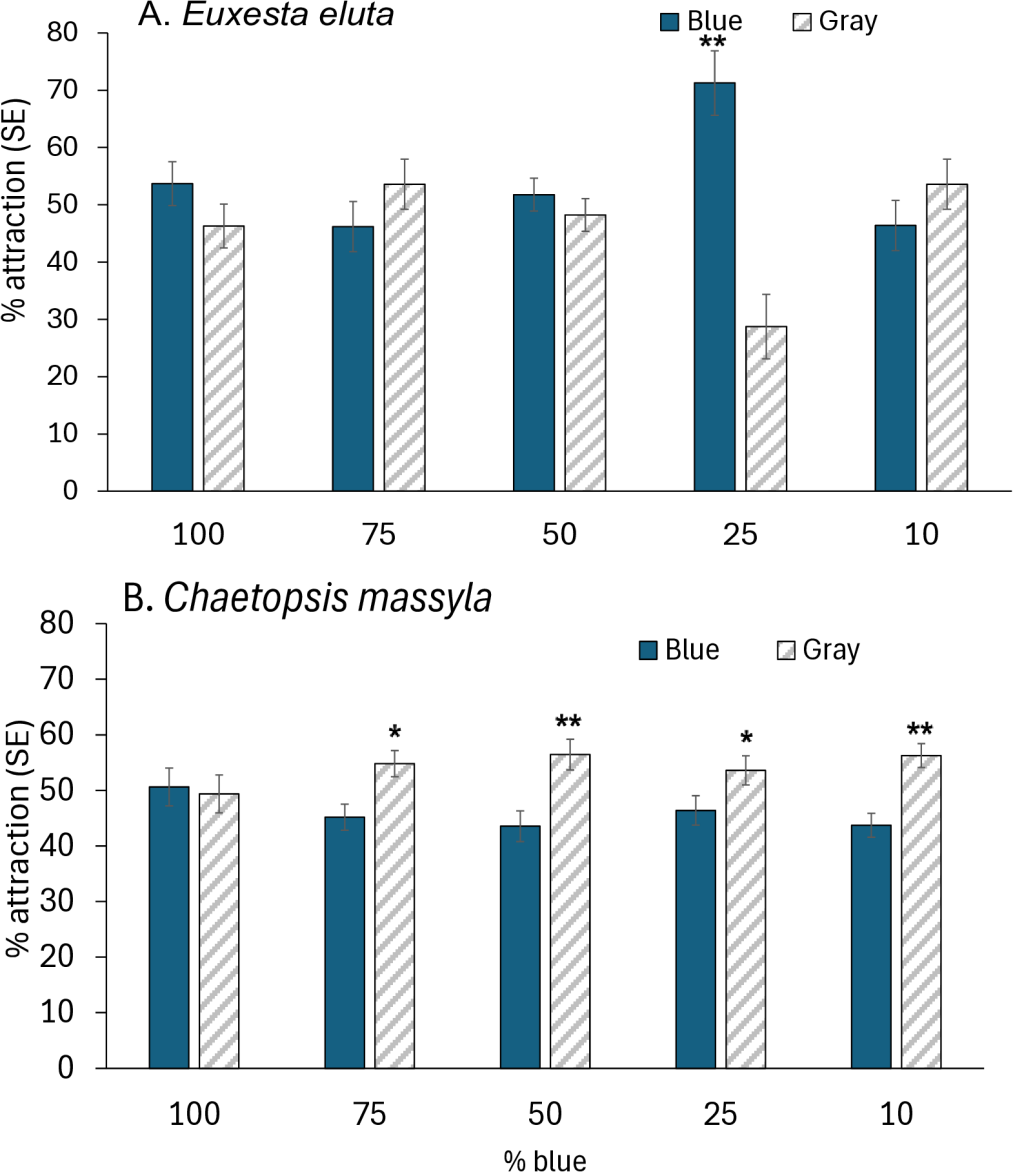
Attraction of *Euxesta eluta* (A) and *Chaetopsis massyla* (B) in paired comparisons with a blue card and a gray card matching in brightness. The different levels of brightness of blue are represented by a range of blue pigment (10–100%) combined with white pigment. Gray cards produced by combination of black and white pigment matched each blue mixture in brightness. Asterisks (*) above paired columns denote statistically significant differences at *P* < 0.05, double asterisks (**) denote significant differences at *P* < 0.001 and no asterisks represent no significant differences. N = 16.

In the assays comparing responses to yellow and gray cards, attraction of *E. eluta* to yellow cards was moderate (46.5–63.4%) with no differences in attraction to the different intensities of yellow (*F* = 1.79, df = 4, 79; *P* = 0.139) (Fig. 6A). For *E. eluta*, attraction to yellow over gray cards was only highly significantly greater for the 100 and 10% yellow mixtures, moderately significant for the 80% yellow mixture and not different from gray for the 20 and 40% yellow mixtures. For *C. massyla*, attraction to yellow cards was strong (65.6–72.1%) and did not differ between intensities of yellow (F = 1.468, df = 4, 79, *P* = 0.22) (Fig. 6B). Attraction of *C. massyla* was significantly greater (*P* < 0.0001) to yellow cards than to gray cards for at all intensities tested (100%, 80%, 40%, 20% and 10% yellow). (Fig 6B). For *E. eluta,* response to green cards was moderately low (45.4–53.07%) with no difference in responses to different intensities (*F* = 0.48, df = 4, 79, *P* = 0.74) (Fig 7A). Response to each brightness level of green was similar to the matching brightness gray card (*P* > 0.05). Attraction of *C. massyla* to green was high (59.6–66.5%), and also with no difference in attraction between different brightness levels of green (*F* = 1.70, df = 4, 79, *P* = 0.158) (Fig 7B). In all comparisons of different brightness levels of green, the attraction of *C. massyla* was greater (*P* < 0.0001) to green cards compared to the matching brightness gray cards. For *E. eluta* responses to blue cards varied from 46.2–71.2% with significant differences in attraction to different brightness levels (*F* = 4.92, df = 4, 79, *P* = 2.49) (Fig 8A). Attraction to 25% blue cards was significantly higher than to the corresponding brightness gray cards (*P* < 0.0001), however responses were similar between blue and gray cards at all other brightness levels (*P* = 0.05). For *C. massyla*, attraction to different brightness levels of blue ranged from (43.7–50.6%) with no significant difference in attraction between the different brightness levels of blue (*F* = 1.18, df = 4, 79, *P* = 0.32) (Fig 8B). Attraction to blue cards was significantly lower than to the gray cards at 10 and 50% blue (*P* < 0.0001), moderately lower for 25 and 75% blue (*P* < 0.01) and equal to gray cards at 100% blue. As controls, comparisons were made between two cards with mid-brightness (15%) gray and there were no differences in response between the treatment and control cards for *C. massyla* (*t* = 0.58, df = 1, 30; *P* = 0.28) and *E. eluta* (*t* = 0.84, df = 1,30; *P* = 0.20).

## Discussion

Both species of cornsilk flies exhibited two broad spectral sensitivity peaks in the UV (350 nm) and green (520–550 nm) region of the spectrum based on the electroretinograms which record physiological responses across multiple retinular cells. These curves were broadly similar between the two cornsilk fly species and to those reported for other flies [16,27,28] and represent the contribution of individual photopigments located in the retinular cells. Pigment absorption templates based on rhodopsin [24,25] provide insight into possible contributions by photopigments with different λ_max_ [29–31]. Our study indicated contributions from UV (355 nm), blue (430 nm), green (500 nm) and yellow green (560 nm) photopigments with slightly higher presence of blue pigments in *E. eluta* compared to *C. massyla*. Through electrophysiological studies in tephritids, responses to UV, blue and green wavelengths have also been reported in tephritids such as *Toxotrypana curvicauda* Gerstaeker (only blue and green [32], *Dacus oleae* (Rossi) [27], and *Bactrocera dorsalis* (Hendel) [33]. While photopigments with different peaks of sensitivity provide visual sensitivity to these wavelengths, not all are associated with specific tactic behaviors, but instead may provide context for other visual processes such as motion detection, background contrast, or color opponency [14,34].

The absorption spectra of visual pigments provide the framework of understanding the spectral sensitivity of the flies, however, other factors such as additional filtering and screening pigments may provide spectral tuning of the spectral sensitivities [13,35,36]. For instance, sensitivity in the higher wavelengths of the spectral sensitivity curve may indicate the presence of screening pigments that result in long wavelength shifting [37,38]. Such pigments are responsible for the typical red color of fly eyes and are present in both species of cornsilk flies.

Perception of UV is widespread across insects and can influence phototaxis, negative phototaxis, phototropism, attraction behaviors as well as detection of open sky or objects against the sky [12,13]. UV plays a role in attraction or repellency for a range of phytophagous insects [12,39]. While UV reflectance from heads and wings of *Anastrepha* and *Ceratitis capitata* (Wiedemann) has been suggested to play a role in mating [40], and it has been suggested that attraction to some traps may be due to UV reflectance [41], definitive roles of UV in tephritid behavior remains less clear. Though UV perception by cornsilk flies is evident from this study, evaluation of its role in behavior will be the basis of future studies.

The behavioral preference for yellow over green by many herbivorous insects has been attributed to yellow as representing a super-normal stimulus due to the higher reflectance of yellow within the green spectral domain [31,42]. An opponent color mechanism related to the proportion of reflectance in the longer wavelengths (green/yellow) compared to the lower wavelengths (blue) is also considered to play a role in these responses [39]. For *E. eluta*, the detection of yellow by the 560 nm photopigment in comparison to the 500 nm photopigment may contribute to the differences in response to yellow and green cards.

Both species of cornsilk fly differentiated yellow hues from the corresponding gray cards indicating both perception of the chromatic cues and associated behavioral response to at least some levels of brightness of yellow. For *E. eluta* yellow cards were more attractive than their paired brightness-matched gray cards but only for the brighter (higher % of white) or darker (more saturated) yellow cards. Presumably mid-bright yellow was perceived but attraction did not occur in response to the color. The stronger attraction of *C. massyla* to yellow cards (10–100%) compared to gray cards indicated that yellow was perceived independent of brightness and that attraction was strongly associated with the color. Both species have been reported to be attracted to yellow in the laboratory [8] and have been collected in yellow, green and white traps [6].

Attraction to green differed between the species. The same level of attraction of *E. eluta* to green or gray cards matched in brightness could indicate that the color was not perceived, however that is unlikely as detection of yellow and green utilizes the same broad-based photoreceptors. More likely the lack of differentiation reflects the lack of behavioral attraction associated with the green color. The difference in response by *E. eluta* to yellow (attraction) and green (non-attraction) infers the ability to detect yellow from green which could be facilitated by the presence of the longer wavelength (560 nm) from the pigment matching nomograms. For *C. massyla*, however, attraction to all brightness levels of green cards over the matching gray cards was consistently strong over the range of 10–100%, indicating that color was the major factor in attraction. In laboratory comparisons, *C. massyla* was most strongly attracted to both yellow and yellow green traps with lower attraction to orange or blue traps [8]. As green is a prevalent background color for most herbivorous insects, perception of the color but lack of attraction to the color is not uncommon and could serve to enhance contrast and perception of other colors against the background or motion detection [13,43]. Kelber [44] proposed that the preference by herbivores for green leaves can be explained by a single opponent interaction between green and blue receptors. Additionally, colors reflecting yellow that are often preferred over green were suggested to possibly be due to the high reflectance in the green portion of the spectrum where yellow would appear [44].

Blue cards elicited differences in attraction responses between *E. eluta* and *C. massyla*. Attraction of *E. eluta* to blue or matching gray cards was similar except for the 25% or mid-tone blue which was significantly more attractive than the gray card of corresponding brightness. In a previous study, both light and mid-tone blue were more attractive to *E. eluta* than yellow in paired laboratory comparisons [8]. Stronger attraction by *Euxesta* flies to mid-blue traps compared to other colored traps including yellow was also reported by Castillo et al. [19]. Responses of *C. massyla* were consistently lower for 10–75% blue mixtures compared to brightness matched grays indicating that blue was perceived but was visually repellent at all levels of brightness. Attraction of light, mid and dark blue were less attractive than yellow for *C. massyla* in paired comparisons in a laboratory study [8]. A blue photoreceptor with λ_max_ at 430 nm could account for the broad shoulder of the spectral sensitivity curves from both species with a higher presence of blue photopigment in *E. eluta* compared to *C. massyla.* Responses to blue differ within Tephritids, with repellency for *B. dorsalis* [32], strong attraction for *Bactrocera tyroni* (Froggatt) [45] and moderate attraction for several *Anastrepha* and *Ceratitis* species [46,47].

Comparison of attraction of insects to color (hue) independent of brightness has been used to provide evidence of color discrimination in *D. oleae* [48], *Lucilia cuprina* (Wiedermann) [49], and *Drosophila* [50]. For some tephritid flies, brightness or intensity contrast against a background plays a more important role than color in attraction [51,52]. However, for most species, color is important in attraction with differences between species. Color for these species is often representative of fruit or foliage. Studies using behavioral assays reported attraction to yellow or yellow-green (*Bactrocera tau* (Walker) [53]; *Bactrocera dorsalis* (Hendel) [18,54–56]; *Anastrepha suspensa* (Loew) [56,57]; *Ceratitis capitata* [57]), red (*B. dorsalis* [55]; *A. suspensa*, [55]; *Rhagoletis pomonella* (Walsh) [58]), green (*Rhagoletis completa* (Cresson) [59]; *Bactrocera* *cucurbitae* (Coquillet) [60], *Bactrocera minax* (Enderlein) [18,61]) and orange (*Anastrepha suspensa (*Loew) [55]; *B. oleae* [62]). Attraction to color may also differ with behavior exhibited, for instance *B. dorsalis* prefers green when feeding and yellow when ovipositing [63]. Additionally, color may be associated with specific behaviors such as landing, oviposition, detection of food sources, motion detection, phototaxis and mate choice and proboscis extension [12,59,64,65].

As members of the family Ulidiidae and the superfamily Tephritoidea, cornsilk flies bear similarities with many other well-known tephritid pests in which visual stimuli have been utilized in the development of different visual traps important for surveillance and control [53,57,60,62]. Color presumably plays a role in host plant location critical for behaviors such as mating and oviposition by cornsilk flies. Understanding the role of color in the behavior of cornsilk flies and inherent similarities and differences in the attraction responses between *E. eluta* and *C. massyla* are important for development of visual traps. Yellow appears to be a common attractive color for attracting both species, however the brightness of yellow is an important factor in attraction. While both species may be attracted to a yellow trap to some degree, attraction and thus trap collection are likely to be more skewed towards *C. massyla*. In contrast, use of mid-tone blue for trapping may be useful for targeted trapping of *E. eluta* with little representation of *C. massyla*.

## Supporting information

S1 Table Percentage of flies in each two-choice assay cage that were collected on either the treatment (colored) or gray card. % colored pigment represents the composition of the treatment which consisted of colored pigment combined with white pigment.
(DOCX)
