# Supplementary material for: Spectral sensitivity and color discrimination of Euxesta eluta and Chaetopsis massyla (Diptera: Ulidiidae)
Source: PLoS One. 2026 Apr 3;21(4):e0346423. doi: 10.1371/journal.pone.0346423 (PMC13048431; doi:10.1371/journal.pone.0346423)
Supplement: S1 Table — % colored pigment represents the composition of the treatment which consisted of colored pigment combined with white pigment. (DOCX) [file pone.0346423.s001.docx]

S1 Table. Percentage of flies in each two-choice assay cage that were collected on either the treatment (colored) or gray card. % colored pigment represents the composition of the treatment which consisted of colored pigment combined with white pigment.

| Paint color | % colored pigment | % responding ± SEM | |
| --- | --- | --- | --- |
|  |  | *Euxesta eluta* | *Chaetopsis massyla* |
| Yellow | 100% | 34.13 ± 3.40 | 80.79 ± 3.12 |
|  | 80% | 26.49 ± 3.06 | 70.62 ± 2.14 |
|  | 40% | 32.25 ± 2.70 | 88.15 ± 2.05 |
|  | 20% | 23.08 ± 2.13 | 84.04 ± 3.17 |
|  | 10% | 38.11 ± 3.25 | 68.21 ± 4.15 |
|  |  |  |  |
| Green | 100% | 36.37 ± 3.18 | 60.45 ± 3.72 |
|  | 70% | 34.04 ± 3.38 | 68.67 ± 2.51 |
|  | 50% | 26.70 ± 1.92 | 68.86 ± 1.95 |
|  | 20% | 33.96 ± 3.09 | 77.32 ± 2.91 |
|  | 10% | 34.92 ± 4.35 | 69.80 ± 2.72 |
|  |  |  |  |
| Blue | 100% | 31.32 ± 2.86 | 59.79 ± 2.23 |
|  | 75% | 42.05 ± 2.24 | 66.26 ± 2.44 |
|  | 50% | 38.92 ± 2.67 | 62.89 ± 3.04 |
|  | 25% | 27.43 ± 2.74 | 58.16 ± 2.79 |
|  | 10% | 51.98 ± 2.52 | 76.53 ± 1.92 |
